# Evidence of structural genomic region recombination in Hepatitis C virus

**DOI:** 10.1186/1743-422X-3-53

**Published:** 2006-06-30

**Authors:** Juan Cristina, Rodney Colina

**Affiliations:** 1 Laboratorio de Virología Molecular, Centro de Investigaciones Nucleares, Facultad de Ciencias, Universidad de la República, Iguá 4225, 11400 Montevideo, Uruguay; 2 Department of Biochemistry and McGill Cancer Center, McGill University, Montreal, Quebec, H3G 1Y6, Canada

## Abstract

**Background/Aim:**

Hepatitis C virus (HCV) has been the subject of intense research and clinical investigation as its major role in human disease has emerged. Although homologous recombination has been demonstrated in many members of the family *Flaviviridae*, to which HCV belongs, there have been few studies reporting recombination on natural populations of HCV. Recombination break-points have been identified in non structural proteins of the HCV genome. Given the implications that recombination has for RNA virus evolution, it is clearly important to determine the extent to which recombination plays a role in HCV evolution. In order to gain insight into these matters, we have performed a phylogenetic analysis of 89 full-length HCV strains from all types and sub-types, isolated all over the world, in order to detect possible recombination events.

**Method:**

Putative recombinant sequences were identified with the use of SimPlot program. Recombination events were confirmed by bootscaning, using putative recombinant sequence as a query.

**Results:**

Two crossing over events were identified in the E1/E2 structural region of an intra-typic (1a/1c) recombinant strain.

**Conclusion:**

Only one of 89 full-length strains studied resulted to be a recombinant HCV strain, revealing that homologous recombination does not play an extensive roll in HCV evolution. Nevertheless, this mechanism can not be denied as a source for generating genetic diversity in natural populations of HCV, since a new intra-typic recombinant strain was found. Moreover, the recombination break-points were found in the structural region of the HCV genome.

## Background

Hepatitis C virus (HCV) is estimated to infect 170 million people worldwide and creates a huge disease burden from chronic, progressive liver disease [1]. HCV has become a major cause of liver cancer and one of the commonest indications of liver transplantation [2,3]. HCV has been classified in the family *Flaviviridae*, although it differs from other members of the family in many details of its genome organization from the original (vector-borne) members of the family [1]. Like most RNA viruses, HCV circulates *in vivo *as a complex population of different but closely related viral variants, commonly referred to as a quasispecies [4-7].

HCV is an enveloped virus with an RNA genome of approximately 9400 bp in length. Most of the genome forms a single open reading frame (ORF) that encodes three structural (core, E1, E2) and seven non-structural (p7, NS2-NS5B) proteins. Short unstranslated regions at each end of the genome (5'NCR and 3'NCR) are required for replication of the genome. This process also requires a *cis*-acting replication element in the coding sequence of NS5B recently described [8]. Translation of the single ORF is dependent on an internal ribosomal entry site (IRES) in the 5'NCR, which interacts directly with the 40S ribosomal subunit during translation initiation [9].

Comparison of nucleotide sequences of variants recovered from different individuals and geographical regions has revealed the existence of at least six major genetic groups [1,10-12]. On the average over the complete genome, these differ in 30–35% of nucleotide sites. Each of the six major genetic groups of HCV contains a series of more closely related sub-types that typically differ from each other by 20–25 % in nucleotide sequences [12].

Different genotypes and sub-types seem to correlate differently for susceptibility to treatment with interferon (IFN) monotherapy or IFN/ribavirin (RBV) combination therapy. Only 10–20 % and 40–50 % of individuals infected chronically with genotype 1 HCV on monotherapy and combination therapy, respectively, exhibit complete and permanent clearance of virus infection. These rates are much lower than the rates of 50 and 70–80 % that are observed on treatment of HCV genotype 2 or 3 infections [3,13].

Until 1999, there was no evidence for recombination in members of the family *Flaviviridae*, although the possibility was considered [14-16]. Accordingly, the vast majority of work on members of this family, including vaccine studies and phylogenetic analyses in which genotypes were identified and sometimes correlated with disease severity, has rested on the implicit assumption that evolution in the family *Flaviviridae *is clonal, with diversity generated through the accumulation of mutational changes [17-19].

This assumption have shown to be invalid, as homologous recombination has been demonstrated in pestiviruses,(bovine viral diarrhoea virus) [20], flaviviruses (all four serotypes of dengue virus) [21-24], hepaciviruses (GB virus C/hepatitis G virus) [25], Japanese encephalitis or St Louis encephalitis virus [26].

Recombination plays a significant role in the evolution of RNA viruses by creating genetic variation. For example, the frequent recovery of poliovirus that result from recombination has the potential to produce "escape mutants" in nature as well as in experiments [27].

Recombination has also been detected in other RNA viruses for which multivalent vaccines are in use or in trials [21,24,28]. The potential for recombination to produce new pathogenic hybrid strains needs to be carefully considered whenever vaccines are used or planned to control RNA viruses. Assumptions that recombination either does not take place or is unimportant in RNA viruses have a history of being proved wrong [24].

Recently, a natural intergenotypic recombinant (2k/1b) of HCV has been identified in Saint Petersburg (Russia) [29,30]. Phylogenetic analyses of HCV strains circulating in Peru, demonstrated the existence of natural intra-genotypic HCV recombinant strains (1a/1b) circulating in the Peruvian population [31].

Given the implications that recombination has for RNA virus evolution [24], it is clearly important to determine the extent to which recombination plays a role in HCV evolution.

## Results

### Phylogenetic profile analysis of full-length HCV strains

**Figure 1:**
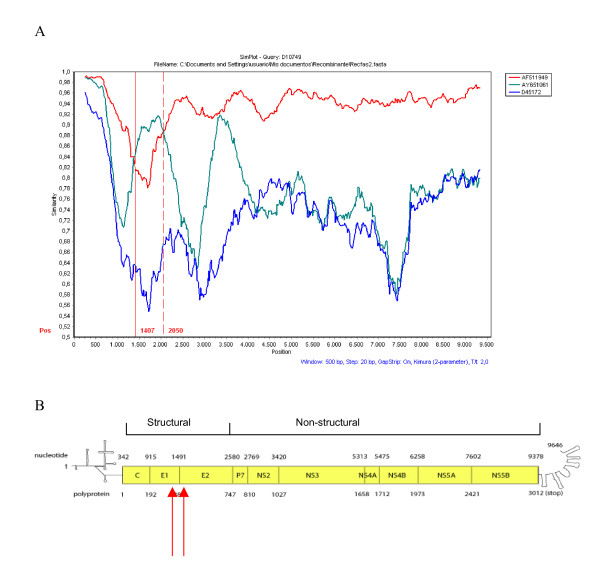
**Phylogenetic profiles of HCV sequences**. In (A) results from SimPlot analysis are shown. The *y*-axis gives the percentage of identity within a sliding window of 500 bp wide centered on the position plotted, with a step size between plots of 20 bp. Comparison of HCV strain D10749 with strains AF511949 (sub-type 1A), AY651061 (sub-type 1C) and D45172 (sub-type 2B) is shown. The red vertical lines show the recombination points at positions 1407 and 2050. In (B) a schematic representation of the HCV genome is shown. Structural and non-structural regions of the genome are indicated on the top of the figure. Nucleotide positions are shown by numbers on the upper part of the scheme. Amino acid codon positions are shown by numbers in the lower part of the scheme. No coding regions at the 5' and 3' of the genome are shown by a line. Coding region is shown by a yellow rectangle, showing the corresponding proteins by name. Recombination points are shown by red arrows.

To gain insight into possible recombination events, a phylogenetic profile analysis was carried out using 89 full-length genome sequences from HCV isolates of all types and sub-types (for strain names, accession numbers and genotypes, see Table 1). This was done by the use of the SimPlot program [32]. Interesting, when the analysis was carried out for strain D10749 (sub-type 1A), two different recombination points (detected at positions 1407 and 2050 of alignment) and two putative parental-like strains (AF511949, sub-type 1A and AY651061, sub-type 1C) are observed (see Fig. 1).

**Table 1:** Full-length HCV sequences.

**Name**	**Genotype**	**Accession number**
H77	1a	AF009606
HCV-H	1a	M67463
COLONEL	1a	AF290978
HC-J1	1a	D10749
HCV-1HCV-PT	1a	M62321
HCV-H	1a	M67463
LTD1-2-XF222	1a	AF511948
LTD6-2-XF224	1a	AF511949
HC-J6	1a	D00944
PHCV-1/SF9_A	1a	AF271632
LTD6-2-XF224	1a	AF511950
HEC278830	1a	AJ238830
AB016785	1b	AB016785
M1LE	1b	AB080299
HCV-N	1b	AF139594
MD1-0	1b	AF165045
274933RU	1b	AF176573
HCV-S1	1b	AF356827
HCV-TR1	1b	AF483269
HCV-A	1b	AJ000009
HCV-AD78	1b	AJ132996
HCV-AD78P1	1b	AJ132997
NC1	1b	AJ238800
HCR6	1b	AY045702
HCV-S	1b	AY460204
AY587016	1b	AY587016
N589	1b	AY587844
HC-C2	1b	D10934
JT	1b	D11168
J33	1b	D14484
HPCPP	1b	D30613
HCV-K1-R1	1b	D50480
HCV-K1-R2	1b	D50481
HCV-K1-R3	1b	D50482
HCV-K1-S1	1b	D50483
HCV-K1-S3	1b	D50484
HCV-K1-S2	1b	D50485
HCV-JS	1b	D85516
D89815	1b	D89815
HCV-J	1b	D90208
HEBEI	1b	L02838
HCV-BK	1b	M58335
HPCGENANTI	1b	M84754
HPCUNKCDS	1b	M96362
HCV-N	1b	S62220
HCU16362	1b	U16362
HD-1	1b	U45476
HCU89019	1b	U89019
HPCHCPO	1b	D45172
JK1-full	1b	X61596
D89815	1b	D89815
TMORF	1b	D89872
HCV-O	1b	AB191333
HD-1	1b	U45476
Con1	1b	AJ238799
HCV-L2	1b	U01214
HCV-K1-S2	1b	D50485
HEC278830	1b	AJ238830
HCV-N	1b	D63857
AY051292	1c	AY051292
HC-G9	1c	D14853
AY051292	1c	AY05292
Khaja1	1c	AY651061
pJ6CF	2a	AF177036
MD2A-7	2a	AF238485
JFH-1	2a	AB047639
AY466460	2a	AY746460
MD2B-1	2b	AF238486
MD2b1-2	2b	AY232731
HC-J8	2b	D10988
JPUT971017	2b	AB030907
BEBE1	2c	D50409
VAT96	2k	AB031663
HCVCENS1	3a	X76918
CB	3a	AF046866
K3A	3a	D28917
HCVCENS1	3a	X76918
NZL1	3a	D17763
HCV-Tr	3b	D49374
JK049	3k	D63821
ED43	4a	Y11604
EUH1480	5a	Y13184
SA13	5a	AF064490
6a33	6a	AY859526
EUHK2	6a	Y12083
TH580	6b	D84262
VN235	6d	D84263
JK046	6g	D63822
VN004	6h	D84265
VN405	6k	D84264
KM45	6k	AY878650

**Figure 2:**
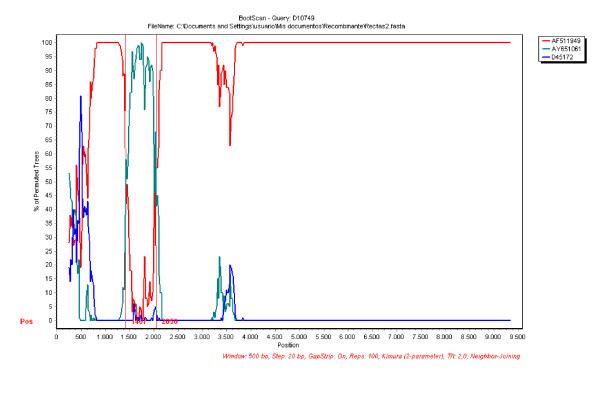
**Bootscanning of HCV sequences**. The *y*-axis gives the percentage of permutated trees using a sliding window of 500 bp wide centered on the position plotted, with a step size between plots of 20 bp. The rest same as Fig.1A.

In order to confirm these results, the same sequences were used for a bootscanning study. The basic principle of bootscanning is that mosaicism is suggested when one observes high levels of phylogenetic relatedness between a query sequence and more than one reference sequence in different genomic regions [33]. When strain D10749 is used as a query, this is observed for this strain and the two putative parental-like strains previously detected (see Fig. 2). The same positions are also observed for the same recombination break-points detected in the similarity index study (see Figs. 1 and 2).

### Profiles of synonymous and non-synonymous substitutions among parental-like and recombinant HCV strains

**Figure 3:**
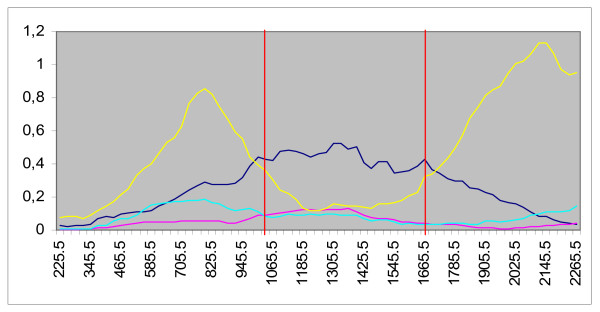
**Profiles of synonymous and nonsynonymous distances of parental-like versus recombinant**. Numbers at the left side of the figure denote distance. Numbers at the bottom of the figure show codon position in the mid point of the window. Comparison AF511949-D10749 is shown in blue and light red for synonymous and nonsynonymous substitutions, respectively. Comparison AY651061-D10749 is shown in yellow and light blue for synonymous and nonsynonymous substitutions, respectively. Vertical red lines show recombination break-points positions.

To gain insight into how the recombination events may have affected the mode of evolution of this HCV isolate, the variation in the rates of synonymous (i.e. no amino acid coding change) and nonsynonymous (i.e. changes in the amino acid coding assignment) substitutions among parental-like and the recombinant HCV strain were calculated for the genome region where the recombination break-points were detected. Synonymous distances are clearly significantly higher than nonsynonymous ones for most of genome region analyzed (see Fig. 3). As a consequence, the ratio of nonsynonymous-to-synonymous amino acid substitutions (*K*_*a*_*/K*_*s*_) is very low for most of this genomic region (see Fig. 3).

Interestingly, the rates of synonymous substitutions in AY651016–D10749 comparison are significantly lower in the region spanned by the recombination break-points, while significantly higher rates are obtained when AF511949–D10749 comparison is performed (see Fig. 3). The results of these studies show that even though recombination took place in the structural region of HCV genome, is has not produced a drastic change in the mode of evolution of the E1/E2 region, since the nonsynonymous substitution rate was maintained at very low rate (see Fig. 3). Thus, at least on this basis, the E1/E2 genomic region does not appear to have been perturbed by the recombination event.

## Discussion

In the present study, analysis of full-length sequences from HCV strains of all types and sub-types provided the opportunity to test the roll that recombination may play in HCV genetic diversity.

The results of this study revealed that recombination may not be extensive in HCV, since from 89 strains studied, recombination was observed in only one case. This is in agreement with the current methodology for HCV genotyping for the vast majority of the cases [10]. Nevertheless, the true frequency of recombination may be underestimated because although there is comparative important number of complete genomes sequences from common genotypes, such as 1b, most studies of HCV variability in high diversity areas are based on analysis of single sub-genomic regions, making detection of potential recombination events unlikely [10].

On the other hand, this study reveals that recombination can not be denied as an evolutionary mechanism for generating diversity in HCV (see Figs. 1 and 2). Moreover, an infectious HCV chimera comprising the complete open reading frame of sub-type 1b strain and the 5'- and 3' non translated regions of a sub-type 1a strain has been constructed and is infectious *in vivo *[34]. A natural inter-genotype recombinant (2k/1b) has been identified in St. Petersburg, Russia [29,30] and a natural intra-typic recombinant (1a/1b) has been identified in Peru [31].

The recombination break-points for non-segmented positive-strand RNA viruses, such as polioviruses and other picornaviruses [35-37] as well as members of the family *Flaviviridae*, are often located in the part of the genome encoding non structural proteins. More recently, recombination break-points have been found in genes encoding structural proteins [38,39]. In the present study, we report recombination events in structural genes (E1/E2 region) between two different sub-types (1a/1c, see Figs. 1 and 2). Recombination may serve two opposite purposes: exploration of a new combination of genomic region from different origins or rescuing of viable genomes from debilitated parental genomes [40].

The recognition of recombination is important not only for unraveling the phylogenetic history of genes, but also for molecular phylogenetic inference. By ignoring the presence of recombination, phylogenetic analysis may be severely compromised [41,42]. For that reason, although recombination may be not appeared to be extensive in natural populations of HCV, this possibility should be taken into account as a mechanism of genetic variation for HCV.

The results of this study, as well as previous ones [29-31] provide evidence that not only does recombination occurs in HCV, but that it occurs in natural populations. In the case of the recombinant described in this study, the distribution of non-synonymous substitutions showed very low rates, revealing that the E1/E2 region of this isolate might have not been perturbed by the recombination events (see Fig. 3). This may also be related to the fact that the differences in this region of the genome among sub-genotypes 1A and 1C, at least in the case of the isolates involved in these studies, are not particularly significant at the amino acid level in the genomic region where the recombination events have occurred.

## Conclusion

Only one of 89 full-length strains studied resulted to be a recombinant HCV strain, revealing that homologous recombination does not play an extensive roll in HCV evolution. A new intra-typic (1a/1c) recombinant strain was found. The recombination break-points were found in the structural (E1/E2) region of the HCV genome. Whether new HCV variants may appear, as a result of recombination events, remains to be established as well as if their fitness permits them to be selected in an HCV population.

## Methods

### Sequences

Full-length genome sequences from 89 HCV isolates where obtained by means of the use of the HCV LANL database [43]. For names, genotypes and accession numbers see Table 1. Sequences were aligned using the CLUSTAL W program [44].

### Recombination analysis

Putative recombinant sequences were identified with the SimPlot program [32]. This program is based on a sliding window method and constitutes a way of graphically displaying the coherence of the sequence relationship over the entire length of a set of aligned homologous sequences. The window width and the step size were set to 500 bp and 20 bp, respectively.

Bootscaning [33] was carried out employing software from the SimPlot program [32], using putative recombinant sequence as a query. Mosaicism is suggested when high levels of phylogenetic relatedness between the query sequence and more than one reference sequence in different genomic regions is obtained.

### Substitution rate analysis

The substitution rate along the open reading frame of the HCV genome, from position 1 to 2490 (relative to the first coding position of strain D10749), was measured using a sliding window method according to the procedure implemented by Alvarez-Valin [45]. Pairwise nucleotide distances (synonymous and nonsynonymous) within each window were estimated by the method of Comeron [46] as implemented in the computer program k-estimator [47]. The window had a size of 150 codons and a movement of 10.

## Competing interests

The author(s) declare that they have no competing interests.

## Authors' contributions

JC and RC conceived, designed and performed the analysis. JC wrote the paper.
